# RETRACTION: Methylation‐Mediated miR‐214 Regulates Proliferation and Drug Sensitivity of Renal Cell Carcinoma Cells Through Targeting LIVIN

**DOI:** 10.1111/jcmm.70583

**Published:** 2025-05-09

## Abstract

**RETRACTION**: H. Xu, S. Wu, X. Shen, Z. Shi, D. Wu, Y. Yuan, W. Jiang, Q. Wang, Q. Ke, Q. Mao, X. Li, Y. Liu, P. Yuan, Q. Zhang, E. Huang, and X. Chen, “Methylation‐Mediated miR‐214 Regulates Proliferation and Drug Sensitivity of Renal Cell Carcinoma Cells Through Targeting LIVIN,” *Journal of Cellular and Molecular Medicine* 24, no. 11 (2020): 6410–6425, https://doi.org/10.1111/jcmm.15287.

The above article, published online on 12 May 2020 in Wiley Online Library (wileyonlinelibrary.com), has been retracted by agreement between the journal Editor‐in‐Chief, Stefan Constantinescu; The Foundation for Cellular and Molecular Medicine; and John Wiley and Sons Ltd. The retraction has been agreed upon following an investigation into concerns raised by a third party, which revealed inappropriate image duplication and resizing within the article (Figure 4C, 5B and 6B) and between this (Figure 4B) and another article published elsewhere by a different group of authors in a different scientific context. The investigation also revealed missing ethical approval regarding the animal experiments presented in the article. Thus, the editors have lost confidence in the presented data and consider the conclusions of this manuscript substantially compromised. The authors and their institute were informed of the concerns and the decision to retract but they remained unresponsive.

**RETRACTION**: H. Xu, S. Wu, X. Shen, Z. Shi, D. Wu, Y. Yuan, W. Jiang, Q. Wang, Q. Ke, Q. Mao, X. Li, Y. Liu, P. Yuan, Q. Zhang, E. Huang, and X. Chen, “Methylation‐Mediated miR‐214 Regulates Proliferation and Drug Sensitivity of Renal Cell Carcinoma Cells Through Targeting LIVIN,” *Journal of Cellular and Molecular Medicine* 24, no. 11 (2020): 6410–6425, https://doi.org/10.1111/jcmm.15287.

The above article, published online on 12 May 2020 in Wiley Online Library (wileyonlinelibrary.com), has been retracted by agreement between the journal Editor‐in‐Chief, Stefan Constantinescu; The Foundation for Cellular and Molecular Medicine; and John Wiley and Sons Ltd. The retraction has been agreed upon following an investigation into concerns raised by a third party, which revealed inappropriate image duplication and resizing within the article (Figure 4C, 5B and 6B) and between this (Figure 4B) and another article published elsewhere by a different group of authors in a different scientific context. The investigation also revealed missing ethical approval regarding the animal experiments presented in the article. Thus, the editors have lost confidence in the presented data and consider the conclusions of this manuscript substantially compromised. The authors and their institute were informed of the concerns and the decision to retract but they remained unresponsive.

